# Expertise affects representation structure and categorical activation of grasp postures in climbing

**DOI:** 10.3389/fpsyg.2014.01008

**Published:** 2014-09-15

**Authors:** Bettina E. Bläsing, Iris Güldenpenning, Dirk Koester, Thomas Schack

**Affiliations:** ^1^Neurocognition and Action–Biomechanics Research Group, Faculty of Psychology and Sport Science, Bielefeld UniversityBielefeld, Germany; ^2^Center of Excellence-Cognitive Interaction Technology, Bielefeld UniversityBielefeld, Germany; ^3^Research Institute for Cognition and Robotics (CoR-Lab), Bielefeld UniversityBielefeld, Germany

**Keywords:** expertise, mental representation, action activation, categorical knowledge, grasping

## Abstract

In indoor rock climbing, the perception of object properties and the adequate execution of grasping actions highly determine climbers' performance. In two consecutive experiments, effects of climbing expertise on the cognitive activation of grasping actions following the presentation of climbing holds was investigated. Experiment 1 evaluated the representation of climbing holds in the long-term memory of climbers and non-climbers with the help of a psychometric measurement method. Within a hierarchical splitting procedure subjects had to decide about the similarity of required grasping postures. For the group of climbers, representation structures corresponded clearly to four grip types. In the group of non-climbers, representation structures differed more strongly than in climbers and did not clearly refer to grip types. To learn about categorical knowledge activation in Experiment 2, a priming paradigm was applied. Images of hands in grasping postures were presented as targets and images of congruent, neutral, or incongruent climbing holds were used as primes. Only in climbers, reaction times were shorter and error rates were smaller for the congruent condition than for the incongruent condition. The neutral condition resulted in intermediate performance. The findings suggest that perception of climbing holds activates the commonly associated grasping postures in climbers but not in non-climbers. The findings of this study give evidence that the categorization of visually perceived objects is fundamentally influenced by the cognitive-motor potential for interaction, which depends on the observer's experience and expertise. Thus, motor expertise not only facilitates precise action perception, but also benefits the perception of action-relevant objects.

## Introduction

Rock climbing requires a multitude of physical and cognitive abilities, as well as their well concerted interaction. One of them is the ability to perceive properties of climbing holds and to execute adequate grasping actions. In indoor climbing, the athlete's goal is to reach the top of a climbing wall by using specific climbing holds that are arranged as routes of different skill requirements. The shape, orientation and relative position as well as the combination of holds thereby determines the adequate grasp and step techniques. Apprehending climbing holds correctly is crucial for planning corresponding actions, and thereby for optimizing the climber's performance (Boschker et al., 2002). Thus, the ability to assign optimal grasps to the reachable holds is a relevant part of a climber's specific cognitive expertise (Pezzulo et al., 2010).

The cognitive aspects of expertise in the domain of rock or indoor climbing have hardly been investigated. One of the rare experimental studies on cognitive issues of climbing expertise has been conducted by Pezzulo et al. (2010), who investigated the ability of novice and expert climbers to memorize climbing routes from presented photographs. Cognitive performance was measured as number of recalled grips in the correct sequence of a route. The main finding was that expert climbers outperformed novices in recalling the sequence of climbing holds of a difficult climbing route that could only be climbed by experts, but not in recalling an easy route that could be climbed both by experts and novices. The authors argued that having the motor competence to climb a visually perceived route enables climbers to mentally simulate mastering it, and that this motor simulation improves the climbers' recall of the grip sequence. Moreover, as participants were not explicitly instructed to mentally simulate the required climbing actions, it was suggested that visually perceiving a climbing route automatically activates the corresponding action sequence in skilled climbers.

Automatic activation of motor components of grasping actions has also been found in other contexts, for example, when participants had to classify kitchen objects or tools (Labeye et al., 2008), or manufactured and natural objects (Tucker and Ellis, 2001, 2004; Grèzes et al., 2003). Labeye et al. (2008) investigated the processing of object features (kitchen tools vs. do-it-yourself (DIY) tools) and action features (implied actions performed with each tool). Participants had to categorize target pictures as kitchen tools or DIY tools. The targets were preceded by a prime picture either depicting a kitchen tool or a DIY tool. Both the object (same vs. different category) and the action features (similar vs. dissimilar implied action) were independently manipulated between the prime and the target pictures. Object and action features independently led to faster processing if they were from the same category or implied similar actions, respectively, compared to different categories or dissimilar actions. Labeye et al. (2008) argued that perceiving the prime picture automatically activates the motor components of the action that may potentially be performed with regard to the object (see also Ellis and Tucker, 2000; Tucker and Ellis, 2001; Bub and Masson, 2010; Masson et al., 2011).

Studies investigating well-known everyday objects (e.g., kitchen tools) suggest that such object representations are associated with certain grasping actions (e.g., Tucker and Ellis, 2004; Labeye et al., 2008). These associations may emerge as a consequence of associated action experience. To further investigate the role of motor experience for object-based action activations, laboratory training studies have been conducted (Creem-Regehr et al., 2007; Kiefer et al., 2007; Weisberg et al., 2007; Cross et al., 2012; Bellebaum et al., 2013). After a training period in which participants explicitly learnt to use objects in a tool-like manner, the manipulation experience became a part of participants' object representations and were automatically activated when the objects were perceived (e.g., Weisberg et al., 2007).

A sports context provides a suitable scenario for investigating expertise effects in object-related action knowledge. Climbing holds have been artificially designed to be used in indoor climbing and are encountered exclusively in this context. Accordingly, people who do not practice indoor climbing have no manipulation experience with climbing holds, whereas sport climbers who frequently train on indoor climbing walls have a large amount of specific manipulation experience. Yet, climbing holds are objects from which particular manipulation potential might be inferred even by non-climbers based on the perceivable shape-properties of the object. Comparing non-climbers' and climbers' processing of climbing holds therefore provides a suitable scenario to dissociate the role of grasping experience and physical object properties in the representation and processing of grasping actions.

Climbing is not a sport performed under time pressure (except speed climbing; Florine and Wright, 2004). However, automatic activations of single grasping actions are an important aspect of a climber's performance. The immediate activation of a grasping action to a perceived climbing hold might decrease cognitive effort in short-term memory and thus save cognitive resources necessary for further action planning (Spiegel et al., 2013). Besides, the direct activation of a grasping action also allows a quick action execution and may thus prevent high energy costs arising when a climber has to remain in a static position evaluating the next action possibility. Based on these considerations, the present study examines object-related action knowledge (Experiment 1) and automatic action activation (Experiment 2) based on perceived climbing holds.

To investigate knowledge representations of climbing-specific grasping actions, Experiment 1 evaluated the relevant cognitive structures of grasping actions related to typical climbing holds in the long-term memory of climbers and non-climbers via Structure Dimensional Analysis (Schack, 2004; SDA; Schack, 2012). It was expected that climbers, but not non-climbers, would categorize climbing holds according to appropriate grip types (functional features) used in indoor climbing rather than according to other object properties.

Experiment 2 was conducted to clarify whether or not the representational clusters are actually associated with motor components that fit the holds in climbers. A priming paradigm was used with pictures of climbing holds as primes and grasping postures as targets (e.g., Güldenpenning et al., 2011, 2013). Climbers were expected to show different effects than non-climbers. Specifically, climbers but not non-climbers should show a congruency effect, i.e., facilitation by congruent primes and inhibition by incongruent primes (Dehaene et al., 1998).

## Experiment 1: categorization of climbing holds

In indoor climbing, climbers have to manage routes of different difficulty level, which requires a multitude of physical and cognitive skills. Applying adequate grasp techniques to the available climbing holds is one of the crucial tasks in this challenge, as it enables the climber to master the route in a safe and efficient way. Experiment 1 investigated if climbing holds are categorically organized depending on their associated manual actions (i.e., adequate grip types) in the long-term memory of experienced indoor climbers. Structure dimensional analysis (SDA; Schack, 2004, 2012) was applied to reveal the relevant representational structures related in the long-term memory of climbers and non-climbers. It was expected that climbers, but not non-climbers, categorized climbing holds according to the appropriate grip types. Previous studies using SDA showed that experts' cluster solutions referred to functionally structured mental representations of complex movements (Bläsing et al., 2009; Land et al., 2013) or to objects affording similar actions (e.g., Stöckel et al., 2012) representing functional action-based categories of partial actions or objects.

### Methods

#### Participants

Twenty-one participants voluntarily took part in Experiment 1 without any exchange or in exchange for course credit. Ten students of sport science without any experience in indoor or outdoor rock climbing, all from Bielefeld University, Germany, were assigned to the non-climbers' group (two females, all right-handed, mean age 24.0 years, range 23–25). All non-climbers were physically active (eight out of ten participants performed individual sports, four performed team sports). The sports most regularly performed by the participants of the non-athlete group included soccer, basketball, fitness training, and running.

Eleven climbers (two females, all right-handed, mean age 27; range 22–34) were recruited from an indoor climbing area, due to their experiences in climbing (mean climbing experience: 5.3 years of training, 3.4 training sessions per week). Referring to the Union Internationale des Associationes d'Alpinisme's (UIAA) grading system describing the difficulty of the climbing route, participants' indoor climbing skills ranged from 6 to 8 (two participants climbed routes graded up to 8, five participants climbed routes graded 7). Five of the climbers also regularly climbed outdoors, with skills corresponding to routes ranging from 5 to 7. Additionally, five out of the ten climbers regularly performed other sports (mostly mountain sports or running).

All participants reported having normal or corrected-to-normal vision, and were naive with regard to the purpose of the experiment. All participants provided written informed consent before testing started. The single experimental session lasted about 30 min. The experiment was performed in accordance with the ethical standards of the sixth revision of the 1964 Declaration of Helsinki (World Medical Assocition, 2008).

#### Apparatus and stimuli

**Figure 1:**
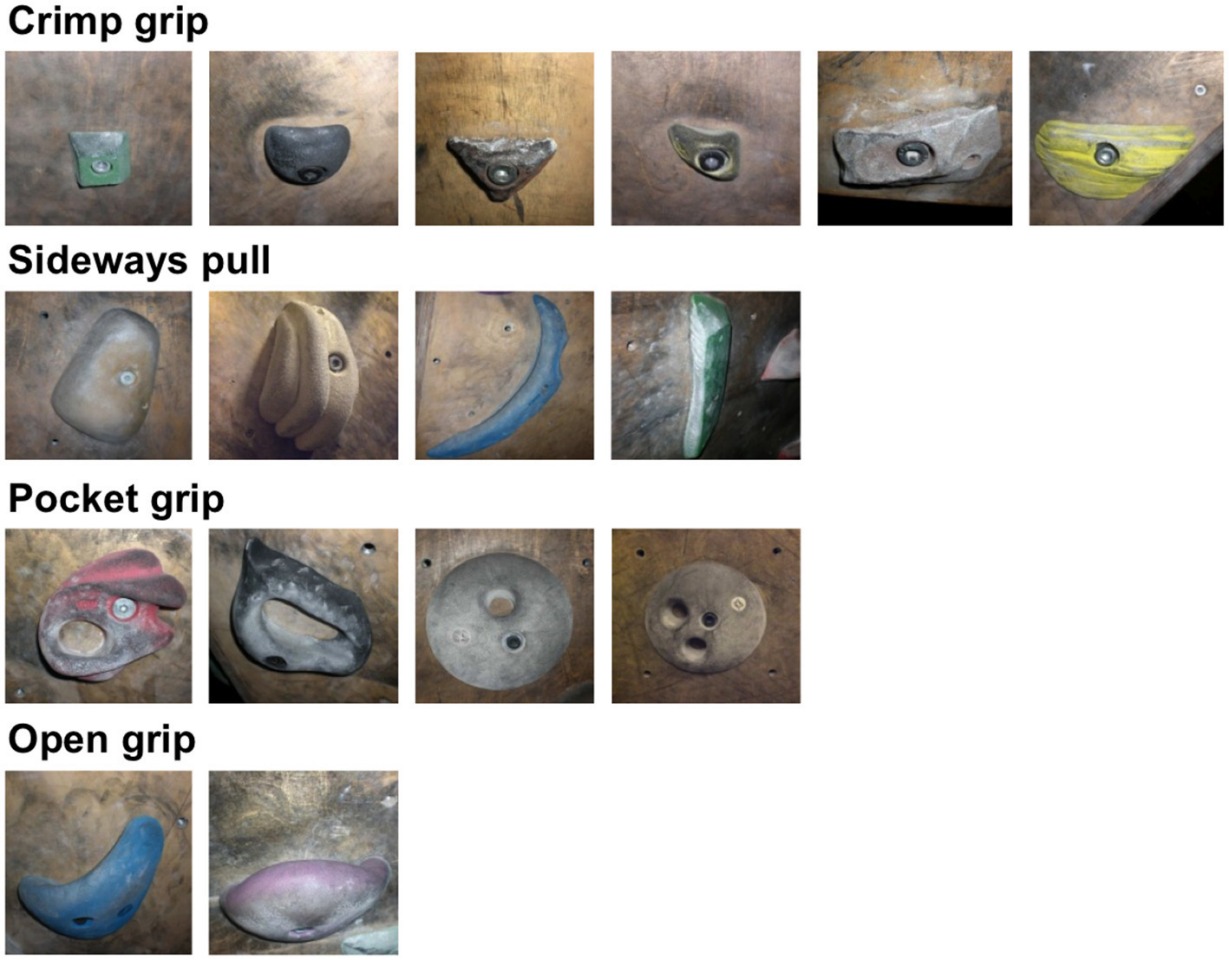
**Stimulus pictures showing climbing holds commonly used in indoor climbing; this grouping of climbing holds according to grip types was used as reference for the participants' results**.

Stimuli consisted of 16 colored photographs of climbing holds of different shapes and sizes, as commonly used in indoor climbing (see Figure 1). The holds were presented to match the climber's perspective, in adequate size relative to each other. All holds were chosen to elicit specific grip types rather than being ambiguous or non-specific. Six out of sixteen holds typically required a crimp grip, four a sideways pull toward the body, four a pocket grip, and two an open grip. This *a priori* attribution of climbing holds to grip types was informed by climbing experts who did not participate in the study and was used as reference for the results of the experiment.

#### Design and procedure

**Figure 2:**
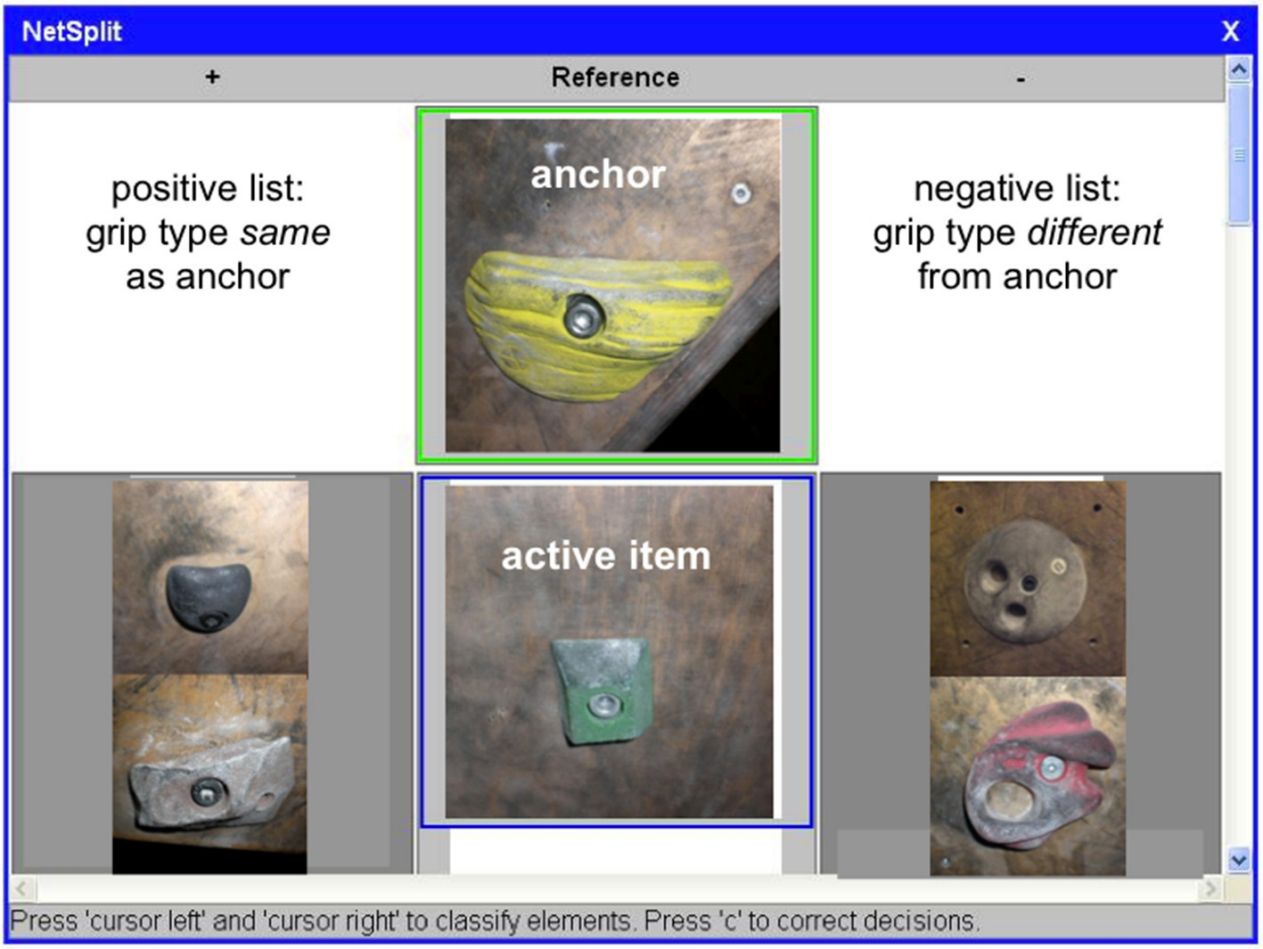
**Presentation of stimuli during the experiment**. The stimulus item in the top position, marked by a green frame, is the current anchor (reference) relative to which the active stimulus below, marked by a blue frame, has to be categorized as affording a similar grip or affording a dissimilar grip. Items appearing in smaller size left and right of the active item have already been assigned to the positive list (similar grips) or to the negative list (dissimilar grips), respectively.

The participants were tested individually while sitting in front of a computer screen. An experimental paradigm named Structure Dimensional Analysis (SDA; Schack, 2004, 2012) was applied to investigate the categorization of climbing holds on the basis of mental representations of specialist grip types in the long-term memory of climbers and non-climbers. SDA was applied via custom-made software (NetSplit). The stimuli were presented in such a way that in each trial, the reference stimulus (or anchor) occurred in the top position marked by a green frame, and the stimulus directly below the reference, marked by a blue frame, had to be assigned by key press to a positive (left) or negative (right) list relative to the reference (see Figure 2). The anchor and the active stimulus picture were presented on the screen with a size of approximately 6 × 6 cm. The participants were instructed to indicate by pressing one of two marked keys if the adequate grasping action directed toward the currently active hold (marked blue) would be of the same type as the one directed toward the climbing hold in the reference position (marked green). Once the response was given, the next trial began, in which the same anchor was presented with another of the remaining items, until all 15 items had been judged as affording a similar or dissimilar grip compared to the anchor; this procedure composed one block. In the next block, a different hold was presented as anchor, in combination with all remaining 15 holds. The whole experiment comprised 16 blocks applied in randomized order, each block with a different item as anchor, resulting in a total of 240 trials.

#### Data analyses

A hierarchical cluster analysis according to SDA (Lander and Lange, 1996; Schack, 2004, 2012) was carried out on the data collected via the previously described splitting procedure in order to obtain mean cluster solutions for the two experimental groups. To achieve this, the sorting task described as part of the experimental procedure was applied to deliver a distance scaling between the items (climbing holds). By this procedure, 16 decision trees were established, as each item occupied once the reference position, resulting in a 16 × 15 matrix of partial quantities in which values took either a negative or positive sign depending on whether the item was judged as belonging to the positive or the negative list relative to the anchor (e.g., if 4 out of the 15 items were assigned to the positive list, these items were each given the value +4, whereas the remaining 11 features assigned to the negative list were each given the value −11). The resulting matrix was then z-transformed for standardization and subsequently transformed into a Euclidian distance matrix as basis for a hierarchical cluster analysis (in accordance with the average-linkage-method). Cluster solutions were determined using a critical Euclidian distance (d_crit_), with all junctures lying below this value forming the apical pole of an underlying concept cluster (for more details on the method, see Schack, 2012). As reference structure, an *a priori* classification of stimulus climbing holds according to specific grip types had been achieved based on interviews with climbing experts who did not participate in the study (see Figure 1). To calculate the similarity between mean group results with the reference structure and to compare each individual participant's cluster solution with the averaged cluster solution of the group and the reference structure (holds 1–6: crimp grip; holds 7–10: sideways pull; holds 11–14: pocket grip; holds 15 and 16: open grip; see also Figure 1), we used the adjusted rand index (ARI; Rand, 1971; Santos and Embrechts, 2009). The ARI provides a measure of similarity on a range between 0 and 1; a score of 0 indicates that two cluster solutions are independent, whereas a score of 1 indicates that two cluster solutions are identical. Scores between these two values indicate the degree of similarity between cluster solutions; the higher the ARI score, the greater is the similarity between the variables.

#### Results

**Figure 3:**
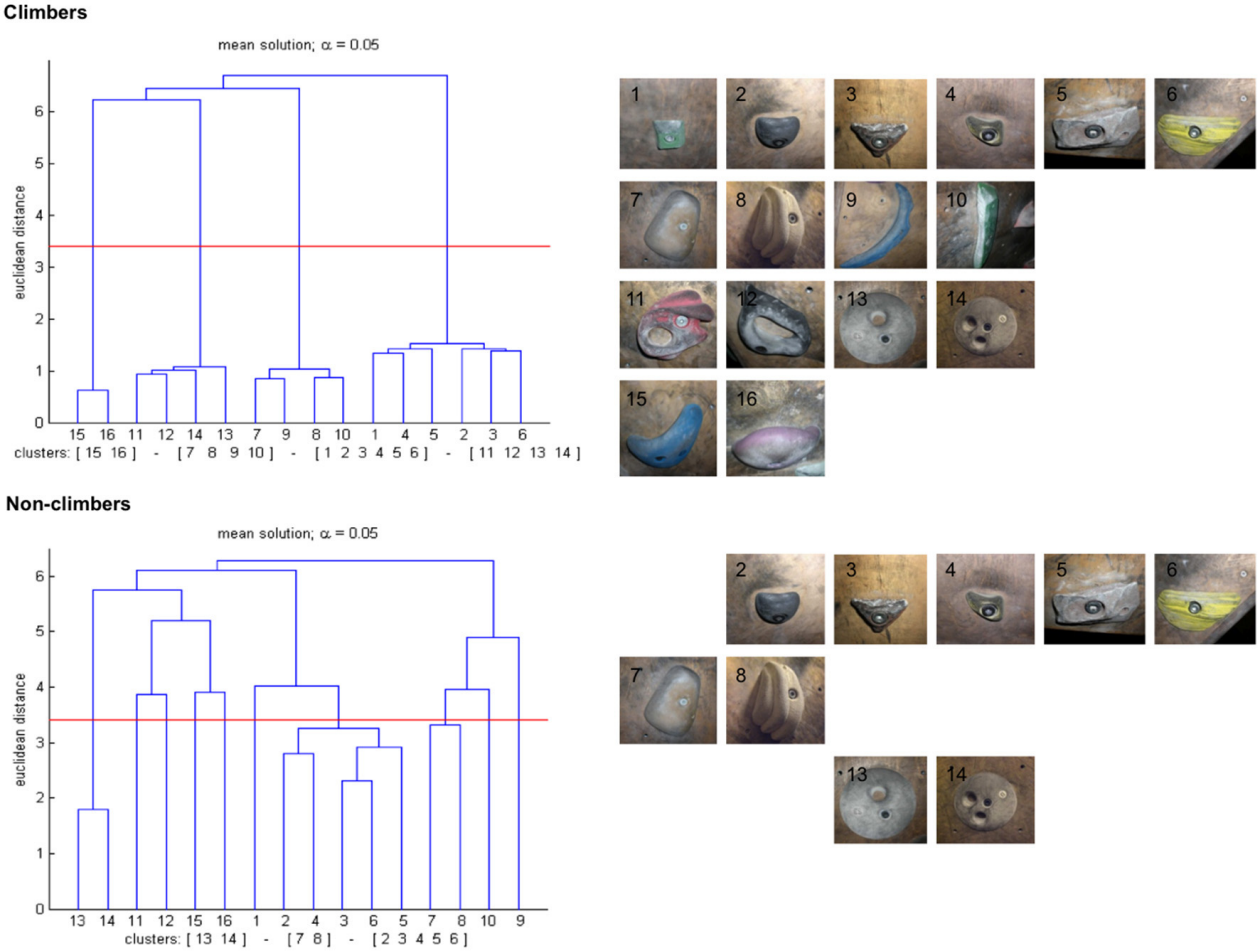
**Results of Experiment 1: mean cluster solutions of groups; top: climbers (*N* = 11); bottom: non-climbers (*N* = 10)**. Left: dendrograms; alpha: 5%, d_crit_ (marked by a horizontal line): 3.4. Numbers on the x-axis refer to the item numbers; numbers on the y-axis indicate Euclidean distance between items according to the cluster analysis via SDA. Right: Stimulus pictures representing cluster solutions.

The hierarchical cluster analysis revealed four clusters corresponding to four grip types for the group of climbers, and three smaller clusters for the non-climbers. The four clusters of the climber group included all 16 holds into clusters that reflected the correct assignment of holds to grip types (cluster 1: items 1–6, cluster 2: 7–10, cluster 3: 11–14, cluster 4: 15 and 16). Euclidean distances between the items of all clusters were all below 1.5 (critical value: 3.4, alpha value: 5%), which reflected a high consistency of the climbers' decisions. The non-climbers' cluster solution contained three clusters, consisting of items 2 to 6, 7 and 8, and 13 and 14. Euclidean distances were all larger than 1.5, and the remaining seven items were singled out (i.e., were not significantly assigned to any cluster). The mean group dendrograms for climbers and non-climbers are presented in Figure 3.

To compare individual participants' cluster solutions to the groups' mean cluster solutions, adjusted rand index (ARI, Santos and Embrechts, 2009) was calculated, which expresses the extent to which the individual cluster solutions differ from the respective averaged group dendrogram. Comparison of the mean group cluster solutions with the reference structure resulted in an ARI score of 1.0 for the climbers (i.e., both cluster solutions were identical) and of 0.535 for the non-climbers. Individual ARI scores of the climbers ranged from 0.685 to 1.0, with a mean of 0.952 ± 0.195 (SD). For the non-climbers, ARI scores were smaller than the climbers' (Mann-Whitney *U*-test; *Z* = −3.887, *p* < 0.001), they ranged from 0.0 to 0.643, with a mean of 0.311 ± 0.206. When non-climbers' ARI scores were calculated with reference to the reference structure, they were also smaller than the climbers' (*Z* = −4.033, *p* < 0.001), ranging from 0.0 to 0.638, with a mean of 0.268 ± 0.194. The non-climbers' ARI scores calculated relative to the group average and the reference structure did not differ (Wilcoxon signed-rank test; *Z* = −1.836, *p* = 0.066).

#### Discussion experiment 1

The hierarchical cluster analysis via SDA revealed four clusters for the group of climbers, and three clusters for the group of non-climbers. For the group of climbers, the mean cluster solution was identical with the functional assignment of grasping holds to grip types (see Figure 1), and individual participants' cluster solutions differed only little from each other; nine out of eleven participants produced a result that was identical with the mean cluster solution. Euclidean distances between items within each cluster were all below 1.5, and thereby small compared to the critical value (*d_crit_* = 3.4). These results point toward a high consistency of decisions made by participants during the experiment, within as well as between participants. These findings suggests that climbers, on the basis of their experience in indoor climbing, could easily associate the presented climbing holds to the corresponding grip types, thereby producing consistent clusters representing functional task-related categories.

In contrast, the non-climbers' mean cluster solution included only nine out of 16 items into clusters, whereas the remaining seven items remained as singletons (that is, these seven items were not categorized by the non-climbers, reflecting a partly non-categorical representation). The three clusters each contained items that belonged to the same grip category. This finding could be explained by two mechanisms: despite their lack of climbing experience, non-climbers might have been able to assign certain climbing holds to appropriate grip types, potentially based on their experience with manipulable objects from a non-climbing context. Non-climbers thereby might have applied the same (or similar) criteria as climbers, but succeeded in doing so only for a subset of the presented items. In this case, the results reflect that attribution to a specific grip type was more difficult for certain climbing holds than for others (e.g., the appropriate grip that required inserting one or more fingers into openings in the hold was apparently easier to recognize for items 13 and 14 than for items 11 and 12).

Alternatively, non-climbers might have grouped items on the basis of other feature-based object similarities related to shape or even color. The latter explanation is supported by the observation that items grouped into the same cluster by the non-climbers looked similar (this is particularly obvious for items 7 and 8 and for items 13 and 14, respectively). Previous studies have shown that novice participants often tend to combine items that show similarity regarding superficial characteristics, rather than task-related functional dependence, into the same cluster (see Schack, 2004). In studies that use partial movements (or basic action concepts, see e.g., Schack and Mechsner, 2006; Schack and Ritter, 2009) in order to investigate mental representations of complex movements, such characteristics often regard the use of body parts (e.g., movement concepts referring to the arms might be combined into one separate cluster, even though this might have no relevance for the functional structure of the movement, as arm movements might have different and even opposed functions during different movement phases). The results suggest that the non-climbers, due to their lack of task-related experience, were less able than the climbers to decide consistently which specific grips were required for the presented climbing holds.

These findings corroborate the notion that the categorization of visually perceived objects is fundamentally influenced by their potential for interaction (i.e., their affordances; Gibson, 1977), the evaluation of which strongly depends on the observer's task-specific experience and expertise. For task-specific objects such as climbing holds, certain features determine their potential use and are, therefore, relevant for functional categorization, whereas other features play a minor role. Climbers, compared to non-climbers, choose more purposefully which of the characteristics of a climbing hold are relevant for the task in question. (In the current study, shape and orientation were task-relevant features, whereas in a different context, the color of climbing holds could be a task-relevant object feature, as it would allow the climber to view the hold as part of a color-coded route).

Taken together, the results of Experiment 1 show that the cognitive representation of objects (i.e., climbing holds) strongly depends on their functional relation to action-based experiences. Based on this finding, we investigated in the subsequent experiment with similar stimuli and two similar groups of participants if and to what extent the perception of task-related objects influences (short-term) processing and the (pre-)activation of object-related actions.

## Experiment 2: cognitive activation of grasping postures

Experiment 2 investigated whether visually perceiving a climbing hold activates the grasping posture commonly associated with this climbing hold. Moreover, it was asked whether the activation of the grasping postures depends on the manipulation experiences with the climbing holds. In a priming experiment, climbers and non-climbers were asked to decide whether a presented target picture reflected a crimp grip or a sideways pull. The preceding prime picture either depicted a climbing hold requiring the grip shown in the target picture (congruent condition; e.g., a hold requiring a sideways pull followed by a sideways pull) or the alternative grip (incongruent condition; e.g., a hold requiring a sideways pull followed by a crimp grip). Moreover, two unspecific conditions were applied. In the positive unspecific condition, the target picture was preceded by a climbing hold which could both be grasped with a crimp grip as well as a sideways pull. In the negative unspecific condition, the preceding climbing hold could neither be grasped with a crimp grip nor with a sideways pull.

It was expected that in experienced climbers a climbing hold would activate the grasping posture commonly associated with the climbing hold, and thus influence responses to the target picture depicting a particular grasping posture (i.e., crimp grip vs. sideways pull). No such activation was expected in participants without manipulation experience with the climbing holds. Moreover, for both groups it was expected that unspecific climbing holds would not activate any grasping posture.

The following three predictions were made: first, a congruency effect was expected for participants with climbing experience; that is, faster response times under conditions in which a climbing hold shown in the prime picture would require the same grasping posture as depicted in the following target picture, and slower response times under conditions in which a climbing hold would require the alternative grasping posture as shown in the target picture. Second, no congruency effect was expected for participants without specific climbing experience. Third, for climbers, response latencies in the unspecific condition were predicted to be in between the congruent and the incongruent condition, whereas for non-climbers, response times should not vary between conditions. The described differences between groups related to the factor congruency are expected to be statistically indicated by an interaction between group and congruency.

### Methods

#### Participants

Thirty two participants voluntarily took part without any exchange or in exchange for course credit. Eighteen students or employees from Bielefeld University, Germany, were assigned to the *non-climber group* (three female, one left-handed, mean age 29.6; range 23–43). Participants of the non-climber group had no experience in climbing. All non-climbers were physically active, performing at least one type of sport (11 participants performed individual sports, 12 performed team sports, 2 performed competitive sports, 2 performed racket sport). Participants of the non-climber group played, for example, soccer, handball, basketball, or regularly performed swimming, running, or fitness training.

Fourteen climbers (one female, two left-handed, mean age 30.0; range 16–43) were recruited from an indoor climbing area for their experiences in climbing (mean training experience: 8.7 years; mean training frequency per week: 4.0 sessions). All recruited climbers were members of the *Deutscher Alpenverein* (German Alpine Association). Referring to the UIAA grading system describing the difficulty of climbing routes, participants' climbing skills ranged between 6 and 10 (two participants were able to climb routes graded 6, two participants climbed routes graded 7, six participants climbed routes graded 8, three participants climbed routes graded 9, and one participant climbed routes graded 10).

All participants reported to have normal or corrected-to-normal vision and were naive with regard to the purpose of the experiment. All participants provided written informed consent before testing started. The single experimental session lasted about 20 min. The experiment was performed in accordance with the ethical standards of the sixth revision of the 1964 Declaration of Helsinki (World Medical Assocition, 2008).

#### Apparatus and stimuli

For stimulus presentation and data collection, a Toshiba Notebook with a 17 inch VGA-Display (vertical retraces 60 Hz) and the software Presentation® (version 14.8) was used. The software controlled the presentation of the stimuli and measured reaction times. Responses had to be given by pressing one of two external response buttons connected via a parallel port with the notebook.

The target pictures were 16 photographs of hands in grasping postures. Eight pictures reflected a crimp grip and eight pictures reflected a sideways pull. Half of the grasping postures reflected a right hand and half of the postures reflected a left hand (images were mirrored) which were used equally often.

**Figure 4:**
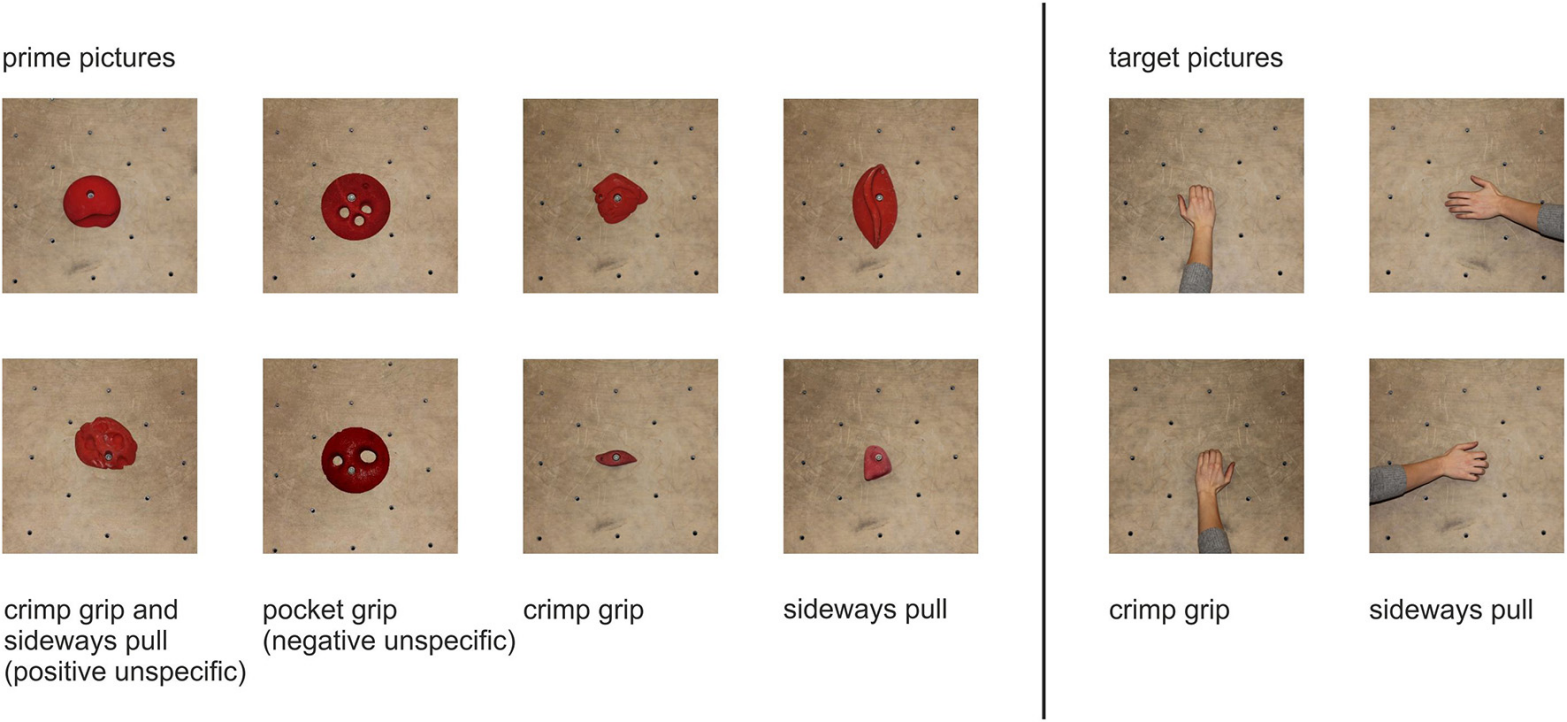
**Examples of the stimulus material used in Experiment 2**. On the left side examples for each type of climbing hold are given. On the right side examples for each type of grasping posture are presented.

As prime pictures, 32 photographs of climbing holds were presented. All climbing holds were red. Four of the climbing holds would require a crimp grip, and four climbing holds would require a sideways pull. Moreover, four climbing holds could be grasped with a crimp grip or a sideways pull (positive unspecific climbing holds). Last, four climbing holds would require a grip that was different from a crimp grip or a sideways pull (negative unspecific climbing hold). The 16 pictures of the climbing holds were mirrored vertically to extend the spectrum of the stimulus material to 32 prime pictures in total. An exemplary illustration of the stimulus material is given in Figure 4.

The background of the climbing holds and of the grasping postures was an indoor climbing wall (see Figure 4). The stimuli had a size of 9.2 × 9.2 cm (346 × 346 pixels). All stimuli were presented centrally on a black background and subtended a visual angle of 8.9° horizontally and vertically from the viewing distance of 60 cm.

#### Design and procedure

The present study used a 4 × 2 mixed factorial design with the within-subject factor *congruency* (congruent condition, incongruent condition, positive and negative unspecific condition) and participants' *expertise* as between-subject factor (climbers vs. non-climbers). The impact of these factors was analyzed with reaction time (RT) and error rate (ER) measures as the dependent variables.

Participants sat in front of a computer screen (60 cm) and were instructed in written form to classify the presented target picture as a crimp grip or as a sideways pull as quickly as possible by pressing one of the two response buttons with the index finger. Moreover, participants were instructed to respond as accurately as possible. The response button assignment was counterbalanced across participants within each group. Before starting the experimental session, each participant performed ten randomized practice trials. Data from this practice block were not analyzed. The following test block consisted of 128 pseudo-randomized prime-target pairs. Each prime picture appeared four times and was either combined with a left hand or right hand crimp grip or with a left hand or right hand sideways pull. The presentation of the order of the prime-target pairs was completely randomized.

**Figure 5:**
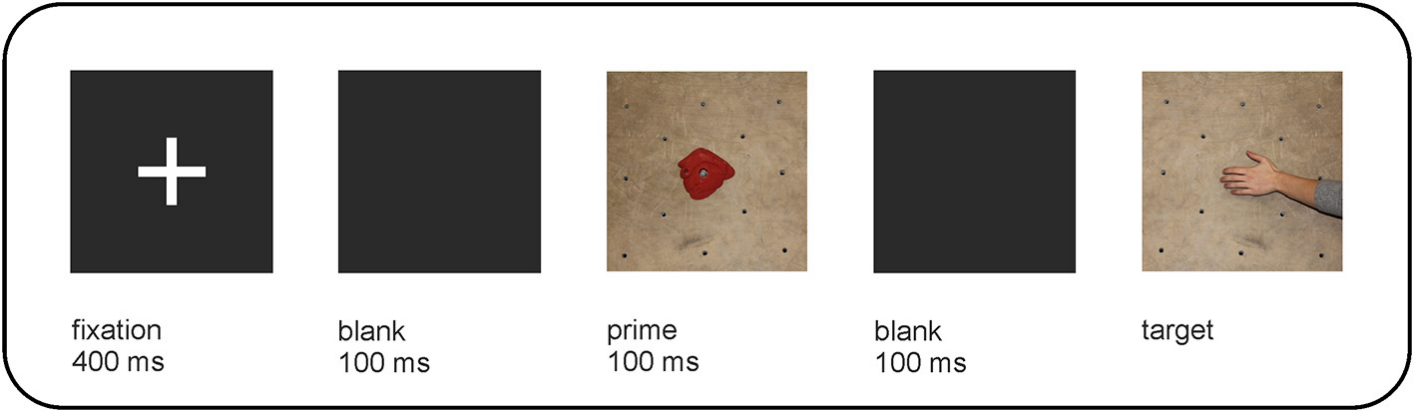
**Stimulus presentation in Experiment 2**. This figure reflects the within-trial procedure for an example of the incongruent condition: a climbing hold requiring a crimp grip is followed by a target picture depicting a sideways pull.

Each trial started with the presentation of a central fixation cross (400 ms), followed by a blank screen (100 ms), the prime (100 ms), a second blank screen (100 ms), and the target (which remained visible on the screen until a response was given). Incorrect responses elicited the word “Fehler” (German for “error”). An inter-trial interval of 1500 ms elapsed before the next trial started. The within-trial procedure is illustrated in Figure 5.

### Results

#### Data analyses

Reaction times (RTs) were screened for outliers using a total cut off. RTs below 200 ms and above 1000 ms were excluded (2.0%). Trials with wrong answers (3.4%) were not used in the analysis of the RTs. The mean RTs from the factorial combinations of the within-subjects factor *congruency* and the between-subjects factor *expertise* were computed for further analysis. A preliminary comparison of the RTs for positive unspecific primes and negative unspecific primes was performed. Separate paired *t*-tests revealed neither a significant difference between positive (547 ms, *s.e.m.* = 21 ms) and negative unspecific primes (552 ms, *s.e.m.* = 22 ms) for climbers, *t*_(13)_ = 0.56, *p* = 0.59, nor for positive (529 ms, *s.e.m.* = 14 ms) and negative unspecific primes (535 ms, *s.e.m.* = 14 ms) for non-climbers, *t*_(17)_ = 0.98, *p* = 0.34. This result indicates that positive unspecific primes and negative unspecific primes did not evoke differential priming effects. Thus, further analyses were computed with the mean value of positive unspecific and negative unspecific primes. This parameter value of the factor congruency was simply termed neutral condition.

Mixed ANOVAs with the within-subjects factor *congruency* (congruent, incongruent, neutral) and the between-subjects factor *expertise* (climbers vs. non-climbers) were performed with RT and ER as dependent variables. A violation of the sphericity-assumption resulted in a correction of the *p*-values according to Greenhouse-Geisser^1^.

#### Reaction times

The within subjects factor *congruency* reached significance, *F*_(1, 60)_ = 4.86, *p* = 0.01, ε = 0.84, η^2^_*p*_ = 0.14, as well as the interaction between *congruency* and *expertise, F*_(2, 60)_ = 3.88, *p* = 0.03, ε = 0.84, η^2^_*p*_ = 0.12. The between subjects factor *expertise* did not reach significance (*p* = 0.75).

To illuminate the source of the interaction, paired *t*-tests were performed separately for climbers (one-tailed) and non-climbers (two-tailed). Climbers responded significantly faster to congruent compared to incongruent prime-target pairs, *t*_(13)_ = 2.52, *p* = 0.01. Responses to grasping postures preceded by a neutral prime were significantly slower than responses to congruent prime-target pairs, *t*_(13)_ = 2.02, *p* = 0.03, and significantly faster than responses to incongruent prime-target pairs, *t*_(13)_ = 1.89, *p* = 0.04.

Non-climbers in contrast responded not differently fast (*p* = 0.72) to grasping postures preceded by a congruent climbing hold and by an incongruent climbing hold. Interestingly, responses to grasping postures preceded by a neutral climbing hold were significantly faster than congruent, *t*_(17)_ = 2.18, *p* = 0.04, and also faster than incongruent climbing holds, *t*_(17)_ = 2.78, *p* = 0.01.

**Figure 6:**
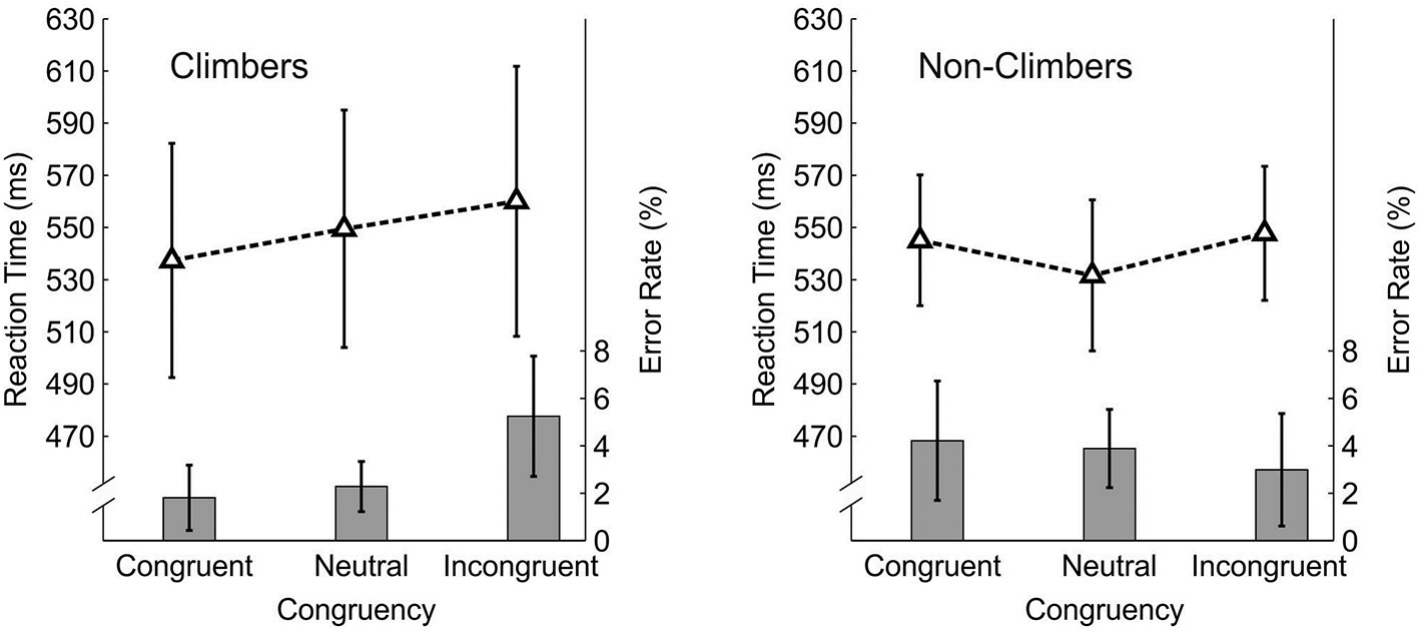
**Results of Experiment 2: Reaction times (RTs) in milliseconds with corresponding 95% confidence intervals (CIs) and error rates (ERs) in percent with corresponding 95% CIs for climbers (left panel) and non-climbers (right panel)**. The dashed lines display RTs scaled on the left vertical axis. The bars illustrate ERs scaled on the right vertical axis. The prime-target conditions are labeled on the x-axis.

Mean values of the RTs and ERs and corresponding confidence intervals are illustrated in Figure 6 and additionally displayed in Data Sheet 1 in the Supplementary Material.

#### Response errors

A mixed ANOVA on the mean ERs neither revealed a significant effect for the within subjects factor *congruency* (*p* = 0.23) nor for the between subjects factor *expertise* (*p* = 0.60). The interaction between *congruency* and *expertise* reached statistical significance, *F*_(2, 60)_ = 4.30, *p* = 0.01, ε = 0.82, η^2^_*p*_ = 0.17. To compare the results of the analysis of the RTs with the ERs, paired *t*-tests were computed separately for climbers (one-tailed) and non-climbers (two-tailed).

For climbers, responding was less error-prone with congruent compared to incongruent trials, *t*_(13)_ = 2.80, *p* = 0.01. The comparison between the incongruent and the neutral condition also reached statistical significance, *t*_(13)_ = 2.8, *p* = 0.01, indicating a higher ER for incongruent compared to neutral prime-target pairs. The comparison between the congruent and the neutral condition did not reach significance (*p* = 0.27).

For non-climbers, none of the comparisons revealed a significant effect (all *p*s > 0.30), but the error rate was slightly smaller for incongruent primes compared to congruent and neutral primes.

#### Discussion experiment 2

Experiment 2 aimed to investigate the activation of grasping postures by visually presenting climbing holds and how such activation is influenced by skill level. In accordance with the hypotheses, a congruency effect was found for climbers, that is, faster responses were found for congruent trials compared to incongruent trials. The inclusion of an unspecific condition revealed that the found congruency effect is based both on speeded responses in the congruent condition and on slowed responses in the incongruent condition compared to the neutral baseline. It would be interesting to determine whether this priming effect arises at the perceptual level (perceptual priming, e.g., Biederman and Cooper, 1992), the cognitive level (e.g., Labeye et al., 2008), or at a motor stage of processing (response priming; e.g., Kunde et al., 2003). Regarding a perceptual locus of the priming effects, there is no visual relation or similarity between the climbing holds and the grasping postures that is larger for the congruent prime target pairs (hold-posture) compared to the incongruent pairs. Hence, we consider it implausible that our priming effects are due to perceptual (dis)similarity of the stimuli. Regarding a potential motor locus, the priming effect is unlikely to reflect a response activation or a response competition effect because the task instruction to classify a grasping posture as a crimp grip or as a sideways pull cannot directly be applied to the climbing hold pictures (primes). That is, the holds themselves should not activate or elicit a response *per se* (i.e., pressing a left or right response button). Therefore, we would argue that the priming effects do not arise during perceptual or motor stages but during cognitive processing stages.

More precisely, the result pattern of speeded responses in congruent trials and slowed responses in incongruent trials, both relative to a neutral baseline, points toward two cognitive processing mechanisms. First, the perception of a given hold leads to an activation of the representation of that hold including the corresponding grasping action. Second, the perception of a given hold seems to lead to a reduced availability of non-corresponding grasping actions. These mechanisms of action activation and action inhibition might help to explain the efficient selection of grasping actions in climbing.

The results found in the non-climbers are also in accordance with the interpretation of a cognitive locus of the priming effects. Participants with no climbing experience are expected to possess no representations of climbing-specific grasping actions. Accordingly, non-climbers did not show any congruency effect. Unexpectedly, however, non-climbers had shorter reaction times for unspecific prime-target pairs compared to congruent und incongruent ones. Besides the possibility that this finding might be a random finding in principle, a possible *post-hoc* explanation for this result could be the following. Round objects similar to the unspecific climbing holds are also common in daily life, for example, as round rotatable button on a washing machine or as knob-like hold of a drawer. It is thus possible that non-climbers applied their general knowledge, that round objects can be grasped either with a crimp grip or with a sideways pull (without knowing these climbing specific concepts), to the predominantly round shapes of climbing holds in the neutral condition. In contrast, non-climbers could not infer any associated grasping action from the unfamiliar climbing holds requiring a crimp grip or a sideways pull. The faster responses to targets following a prime picture with a round hold thus may reflect unspecific activations of grasping representations. Further studies are needed to confirm this suggestion.

## General discussion

This study explored the relationship between the action-based cognitive representations of climbing holds and the object-based activation of the corresponding grasping actions. Experiment 1 investigated the structure of skill representations. (Note that we use the term climbing skill in this context specifically for the skilled manual use of climbing holds, i.e., the knowledge of correct grip application for individual climbing holds.) The results of Experiment 1 suggest that climbers organize visually perceived climbing holds categorically according to functional features (i.e., how to grasp in an indoor climbing context). Non-climbers, in contrast, showed an organizational structure that was not categorical in terms of skill-based (climbing) knowledge but rather based on unspecific world knowledge or superficial object features (e.g., form or color). Experiment 2 investigated the access of grasping knowledge, in particular whether and how functional features (i.e., grasping postures) are activated by object features (i.e., shapes of climbing holds). Here, we found evidence for activation of matching grasping postures and inhibition of non-matching grasping postures by the perception of grip-specific holds.

We argue that in climbers, but not in non-climbers, the categorical memory structure reflects the functional features of climbing holds in the context of indoor climbing. This structure appears to follow functional distinctions of the associated actions and reflects climbers' manifold experiences of task-related action-effect coupling (Hoffmann, 2003). The qualitative changes in memory structures might also change perceptual information processing (cf. Ericsson and Kintsch, 1995). Having command over, for example, a grip category related to sideways pull would facilitate the recognition and processing of potentially distinct holds regarding their applicability and functional relevance for the adequate motor action. The present findings thus provide insights as to how skilled climbers may achieve a better climbing performance: automatic activation of adequate grasping actions in response to the perception of a specific climbing hold can be regarded as crucial mechanism to reduce the cognitive demands involved in decision making and the planning of selected motor actions. This mechanism thereby serves to reduce cognitive processing time, which, importantly in the climbing context, leads to reduced physical energy costs (i.e., muscle force needed to remain in a static posture while evaluating the next move). Climbing thereby represents a relevant example of how skill-based knowledge that can be accessed explicitly for cognitive control also supports evaluative, strategic action planning under resource constrained conditions.

Our results are in accordance with findings by Pezzulo et al. (2010) who reported better recall performance for difficult climbing routes in climbers compared with non-climbers. Pezzulo et al. (2010, p. 72) speculate that experts are better able to form motor chunks that are necessary for mastering perceived climbing routes by means of simulation and hence are better in memorizing the routes, but these authors also consider alternative explanations such as better visual imagery in experts. Whereas Pezzulo et al. (2010) used an offline measure of memorization, Experiment 2 in our study investigated online processing (i.e., short-term activation) of skill representations which are suggested to be categorically structured according to our Experiment 1.

The assumption of categorical skill representations raises the question of how specific actions are selected. Generally, it is conceivable that some holds have multiple grasping possibilities and should, thus, activate more than one category of climbing holds. Here, Experiment 2 yielded evidence for processes of activation and inhibition. The results of Experiment 2 are also in accordance with Labeye et al. (2008) who also found that the perception of (manipulable) objects activates associated actions, even though we used considerably longer stimulus onset asynchronies compared to Labeye et al. (2008). Moreover, Experiment 2 suggests that such action feature activations depend on previous learning or experience with the object and not on the pure physical object properties as the non-climbers' data pattern showed the fastest responses in the unspecific conditions.

Our results corroborate findings from studies of complex movement representations (Bläsing et al., 2009; Güldenpenning et al., 2011, 2013; Weigelt et al., 2011; Land et al., 2013) and skill acquisition (Frank et al., 2013). They emphasize the role of cognitive representations and processes in action control and support the view that skill representations are based on categorical knowledge. In this regard, our study confirms the role of cognitive processes in the control of complex human actions as proposed in frameworks such as the ideomotor approach (Koch et al., 2004; for a histoical overview of the ideo-motor principle, see Stock and Stock, 2004), the theory of event coding (TEC; Hommel et al., 2001), or the cognitive action architecture approach (CAA-A; Schack and Ritter, 2009; Land et al., 2013).

Taken together, the present studies investigated the cognitive representations of indoor climbing holds and the perceptual processing of such holds and associated grasping postures in climbers and non-climbers. Experienced climbers represent holds according to their functions (i.e., grip types) whereas non-climbers show less structure in their representations and organize these according to unspecific action knowledge or superficial features. It was also found that the perception of climbing holds activates the matching grasping posture and inhibits non-matching postures in climbers but not in non-climbers. These findings suggest a processing advantage and mechanism of categorical action representations. Furthermore, the findings show that action experience modifies the relevant object representations by associating action features to the representations of corresponding objects.

### Conflict of interest statement

The authors declare that the research was conducted in the absence of any commercial or financial relationships that could be construed as a potential conflict of interest.

## Footnotes

^1^A violation of the sphericity-assumption requires modifications of the degrees of freedom (*df*s) to reduce the Type I error. The estimates for this modification is denoted by epsilon (ε) providing a measure of deviation from sphericity. An epsilon of 1 indicates that sphericity is exactly met, smaller epsilon values indicate increasing deviation. Due to better reading, the uncorrected *df*s are given for the *F*-value, but the corrected *df*s can be easily calculated by multiplying the original *df*s with ε.
