# Supplementary material for: Expertise affects representation structure and categorical activation of grasp postures in climbing
Source: Front Psychol. 2014 Sep 15;5:1008. doi: 10.3389/fpsyg.2014.01008 (PMC4164095; doi:10.3389/fpsyg.2014.01008)
Supplement: Supplementary file 1 [file DataSheet1.DOCX]

Supplementary Material: Mean reaction times (RT) in milliseconds and mean error rate (ER) as percentages as a function of congruency, and group. The 95% confidence intervals (CIs) of the means are given in parentheses.

|  |  |  | Congruency | | |
| --- | --- | --- | --- | --- | --- |
|  |  |  | congruent | neutral | incongruent |
| Climbers | RT (*CI*) | | 537 (±45) | 550 (±47) | 560 (±52) |
|  | ER (CI) | | 1.8 (±1.4) | 2.3 (±1.1) | 5.2 (±2.5) |
| Non-climbers | RT (*CI*) | | 545 (±25) | 532 (±29) | 548 (±26) |
|  | ER (CI) | | 4.2 (±2.5) | 3.9 (±1.6) | 3.0 (±2.4) |
